# Evaluation of perioperative hypersensitiviy reactions: post-event interaction between anesthesiologist and alergist

**DOI:** 10.1186/1939-4551-8-S1-A143

**Published:** 2015-04-08

**Authors:** Maria Anita Spíndola, Jane Da Silva, Edelton Flávio Morato, Edelton Flávio Morato

**Affiliations:** 1 Hospital Universitário Polydoro Ernani De São Thiago - HU/Ufsc, Brazil; 2 Universidade Federal De Santa Catarina/ Centro De Ciências Biológicas - CCB/Ufsc, Brazil

## Background

Allergic reactions during perioperative period may vary in terms of incidence, type of reaction and severity. Reactions ranging from mild skin disorders up to cardiac arrest might be observed. The lack of post-event investigation and a failure of interaction, due to a poor communication, between anesthesiologist and allergist represent risks for a re-exposure to the allergen. The existence of an instrument to enable allergist and anesthesiologist to exchanging informations about the patient and his allergic reaction can reduce this risk of re-exposition. The aim of the study was to develop a protocol for communication between anesthesiologist and allergist for investigation of perioperative allergic reactions.

## Methods

Consecutive mettings were done including the doctors in charge of the allergy and immunology services and the anesthesiologist from the Núcleo de Avaliação de Reações do tipo Aléricas a Drogas at the Universidade Federal de Santa Catarina (NARTAD-HU-UFSC). Data from literature concerning to perioperative hypersensitivity reactions were searched, foccusing important points to an effective communication between anesthesiologists and allergists during the investigations of allergic reactions.

## Results

A protocol was created using a form in which the first part should be fulfilled by the anesthesiologist, determining the reaction intensity level, all agents involved, required examens and the treatment settled during the perioperative reaction. Additionaly, a copy of the anesthesia record should be included. After, this form must be forwarded to the allergist who should include investigative measures, results of tests and its interpretation.

## Conclusions

Communication between anesthesiologists and allergists for investigation of allergic reactions is essential to prevent or reduce the risk of re-exposure to the causative agent, and also to avoid excluding non implicated agents. An investigative protocol exchangeable between both professionals can be a useful tool for this purpose.

